# Capsaicin Suppresses Cell Proliferation, Induces Cell Cycle Arrest and ROS Production in Bladder Cancer Cells through FOXO3a-Mediated Pathways

**DOI:** 10.3390/molecules21101406

**Published:** 2016-10-21

**Authors:** Kaiyu Qian, Gang Wang, Rui Cao, Tao Liu, Guofeng Qian, Xinyuan Guan, Zhongqiang Guo, Yu Xiao, Xinghuan Wang

**Affiliations:** 1 Department of Urology, Zhongnan Hospital of Wuhan University, Wuhan 430071, China; 2 Department of Urology, The Fifth Hospital of Wuhan, Wuhan 430050, China; 3 Department of Urology, Jingzhou Central Hospital, Jingzhou 434020, China; 4 Department of Endocrinology, The First Affiliated Hospital of Zhejiang University, Hangzhou 310003, China; 5 Department of Clinical Oncology, Li Ka Shing Faculty of Medicine, University of Hong Kong, Hong Kong, China; 6 Center for Medical Science Research, Zhongnan Hospital of Wuhan University, Wuhan 430071, China

**Keywords:** capsaicin, bladder cancer, cell cycle, ROS, migration, xenograft

## Abstract

Capsaicin (CAP), a highly selective agonist for transient receptor potential vanilloid type 1 (TRPV1), has been widely reported to exhibit anti-oxidant, anti-inflammation and anticancer activities. Currently, several therapeutic approaches for bladder cancer (BCa) are available, but accompanied by unfavorable outcomes. Previous studies reported a potential clinical effect of CAP to prevent BCa tumorigenesis. However, its underlying molecular mechanism still remains unknown. Our transcriptome analysis suggested a close link among calcium signaling pathway, cell cycle regulation, ROS metabolism and FOXO signaling pathway in BCa. In this study, several experiments were performed to investigate the effects of CAP on BCa cells (5637 and T24) and NOD/SCID mice. Our results showed that CAP could suppress BCa tumorigenesis by inhibiting its proliferation both in vitro and in vivo. Moreover, CAP induced cell cycle arrest at G0/G1 phase and ROS production. Importantly, our studies revealed a strong increase of FOXO3a after treatment with CAP. Furthermore, we observed no significant alteration of apoptosis by CAP, whereas Catalase and SOD2 were considerably upregulated, which could clear ROS and protect against cell death. Thus, our results suggested that CAP could inhibit viability and tumorigenesis of BCa possibly via FOXO3a-mediated pathways.

## 1. Introduction

Bladder cancer (BCa), also known as urinary bladder cancer, is one of the most common malignant tumors and ranks as the 9th leading cause of death worldwide [1,2]. Besides high morbidity and mortality, BCa is particularly characterized by a high recurrence rate risk [3] and results in enormous burden on patients and health care system [4,5]. To treat bladder cancer, various medical procedures, including transurethral resection of the bladder tumor, radical cystectomy, chemotherapy, immunotherapy, alone or combined, are widely used [6,7,8,9]. However, current therapeutic approaches have a variety of adverse effects on patients, such as local recurrence, distant metastasis, low survival rate and high cost [5,8,10]. Thus, more effective therapies need to be developed for BCa.

Capsaicin (8-methyl-*N*-vanillyl-6-nonenamide) is the main pungent ingredient in *Capsicum* species plants, consumed as a food additive throughout the world for its pungency [11]. Capsaicin (CAP) is a highly selective agonist for the transient receptor potential vanilloid type 1 (TRPV1) [12,13]. In addition to the prototypical function of Ca^2+^ channel, TRPV1 has been described to be correlated with BCa [14] and also revealed as a target for drug development [15,16]. Recently, CAP has been reported for its analgesic, antioxidant, anti-inflammatory, and anticancer activity [16,17]. Moreover, CAP has been suggested a potential clinical significance in tumor therapy [18,19].

Our group has focused on the transient receptor potential family (TRP family) and effects of CAP in urological tumors including bladder cancer [20,21]. Despite recent progress, the exact mechanism of BCa pathogenesis remains largely unknown. Our recent studies based on microarray analysis using human bladder cancer tissues compared with normal bladder tissues (GEO accession number: GSE76211), suggested a close correlation between the calcium signaling pathway, FOXO signaling pathway, cell cycle regulation, PPARγ-related reactive oxygen species (ROS) metabolism and tumorigenesis of BCa [21,22,23]. Furthermore, our previous studies also suggested that CAP could induce cell cycle arrest in human BCa cell line 5637 [24], mediate cell death in mouse BCa cell line MBT-2 [25] and human BCa cell line T24 in vitro [26] as well as inhibit tumor growth in T24-transplated nude mice in vivo [26]. One possible underlying mechanism might be that CAP could affect SIRT1 [17] and ROS production, which is calcium entry dependent [26], and therefore link ROS and BCa cell death together. However, the interpretations from most studies investigating CAP in human bladder cancer were based on a sole cell line, and/or few data from mouse model, lacking detailed genes and pathways related. Therefore, more evidences are needed to clarify the inhibitory effect of CAP on regulation of proliferation, cell cycle and ROS metabolism in bladder cancer both in vitro and in vivo.

## 2. Results

### 2.1. CAP Inhibited BCa Cell Proliferation and Migration

**Figure 1:**
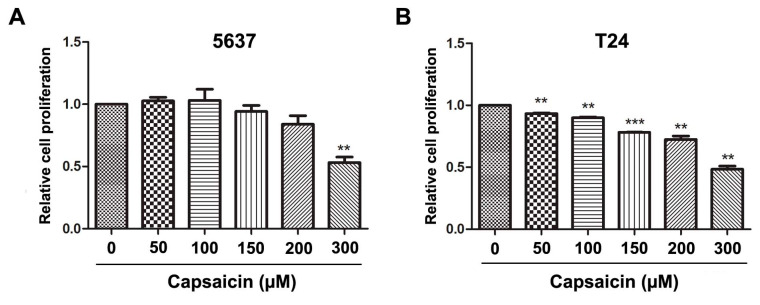

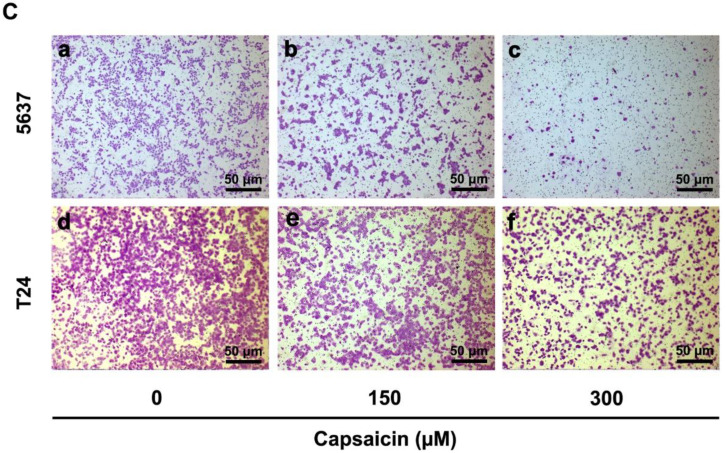

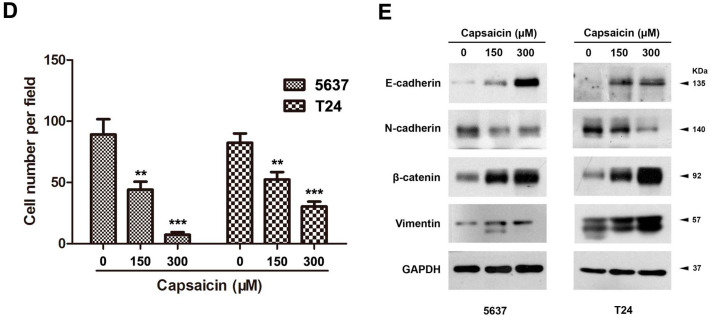
Capsaicin inhibits BCa cell proliferation and migration in vitro. (**A**,**B**) Relative cell proliferation of 5637 and T24 cells treated by CAP at distinct concentrations (0, 50, 100, 150, 200 and 300 µM) for 48 h were measured by MTT assay, to determinate the appropriate concentrations of CAP treatment on 5637 and T24 cells. ** *p* < 0.01, *** *p* < 0.001; (**C**) Transwell migration assay for CAP treated 5637 (a–c) and T24 cells (d–f) at 0, 150 and 300 µM for 48 h. The scale bar for (a–f) is 50 μm; (**D**) Statistical analysis of transwell migration assay, showed significantly reduced migrated cell number of 5637 and T24 cells after CAP treatment at 150 and 300 µM. ** *p* < 0.01, *** *p* < 0.001; (**E**) Western blot analysis for proteins involved in EMT regulation, revealing that *E*-cadherin and β-catenin were strongly increased, in contrast, *N*-cadherin was strongly decreased after CAP treatment, whereas Vimentin was only slightly altered. GAPDH was used as a loading control. Cell types, concentrations of CAP and protein masses were indicated. All data shown were mean ± SD of triplicate measurements and repeated at least three independent experiments with similar results.

To investigate the effects of CAP on cell viability in the BCa cells, 5637 (Figure 1A) and T24 (Figure 1B) cells were treated with CAP at different concentrations (0, 50, 100, 150, 200 and 300 µM) for 48 h. An MTT assay was used to measure the cell viability. The results exhibited a reduced tendency of relative cell proliferation rate in a dose-dependent manner and a significantly reduction in both 5637 and T24 cells at 300 µM. In the following, in vitro studies with CAP at 0 µM (control), 150 µM (moderate dose) and 300 µM (high dose) were done.

Cell migration was measured using transwell migration assay (Figure 1C). After treatment with CAP at 150 and 300 µM for 48 h, migration rates of 5637 cells and T24 cells were significantly decreased (Figure 1D). Furthermore, the levels of proteins (*E*-cadherin, *N*-cadherin, β-catenin and Vimentin) involved in epithelial-mesenchymal transition (EMT) process were altered after treatment with CAP (Figure 1E). The results showed that *E*-cadherin and β-catenin were strongly increased and *N*-cadherin was significantly decreased after CAP treatment at 150 and 300 µM for 48 h, whereas the level of Vimentin was slightly changed.

### 2.2. CAP Induced an Increase of ROS Production in BCa Cells

**Figure 2:**
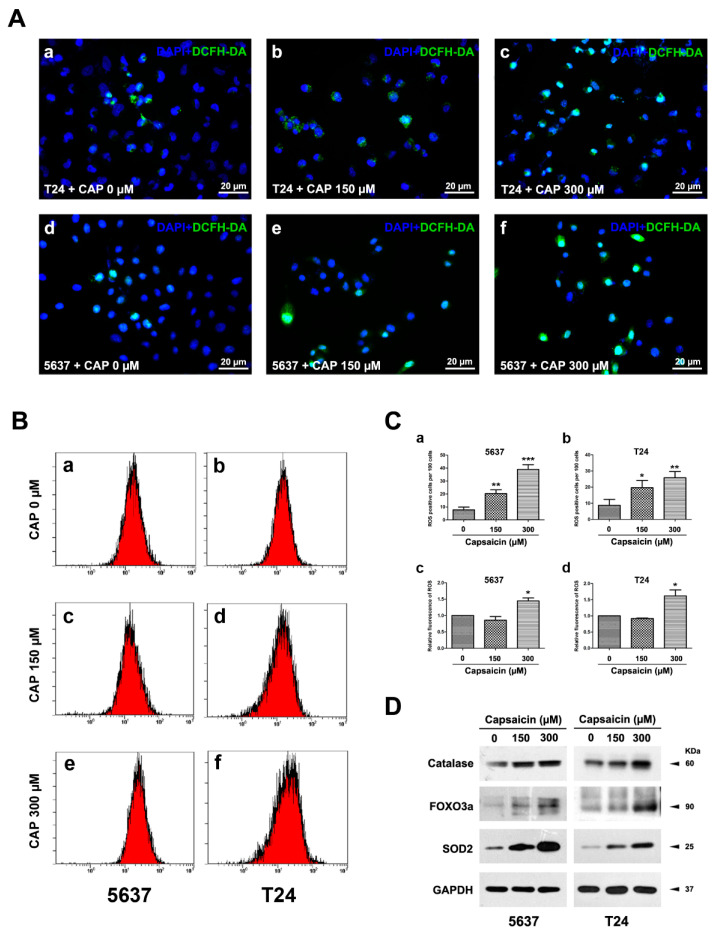
Induction of ROS production and enzymes involved in ROS metabolism by CAP treatment in vitro. (**A**) Representative DCFH-DA staining (green) for ROS production in T24 cells (a–c) and 5637 cells (d–f) treated by CAP at 0, 150 and 300 µM for 48 h. Nuclei were stained by DAPI (blue). The scale bars for (a–f) are 20 μm; (**B**) Representative flow cytometry images for ROS detection by DCFH-DA in 5637 (a,c,e) and T24 cells (b,d,f) by CAP treatment at 0, 150 and 300 µM for 48 h; (**C**) Statistical analysis of cell number with positive DCFH-DA staining (a,b) and relative fluorescence of DCFH-DA using flow cytometry analysis (c,d), indicating significantly increased DCFH-DA positive BCa cells after CAP treatment. Results shown were mean ± SD of triplicate measurements and repeated three independent experiments. * *p* < 0.05, ** *p* < 0.01, *** *p* < 0.001; (**D**) Western blot analysis revealed a strong upregulation of proteins involved in ROS metabolism: Catalase, FOXO3a, SOD2. GAPDH was used as a loading control. Cell types, CAP concentrations and protein masses were indicated.

To investigate the effects of CAP on ROS production in BCa cells, we analyzed cellular ROS levels in 5637 and T24 cells after treatment with CAP at 0, 150 and 300 µM for 48 h by DCFH-DA staining (Figure 2A) and flow cytometry (Figure 2B). Indeed, we observed a clear dose-dependent increase in cellular ROS levels after incubation with CAP by staining (Figure 2C (a,b)). Consistently, the results examined by flow cytometry analysis also showed a significantly higher fluorescence of ROS in 300 µM group than the control group (Figure 2C (c,d)). Moreover, western blot analysis revealed that CAP could induce a strong increase of protein levels for anti-oxidative enzymes (Figure 2D), such as mitochondria superoxide dismutase 2 (SOD2), transcription factor FOXO3a and Catalase, one of the most important antioxidant enzymes, which catalyzes the decomposition of H_2_O_2_ to H_2_O [27].

### 2.3. CAP Triggered Cell Cycle Arrest at G0/G1 Phase, But No Significant Effect on Apoptosis in BCa Cells

**Figure 3:**
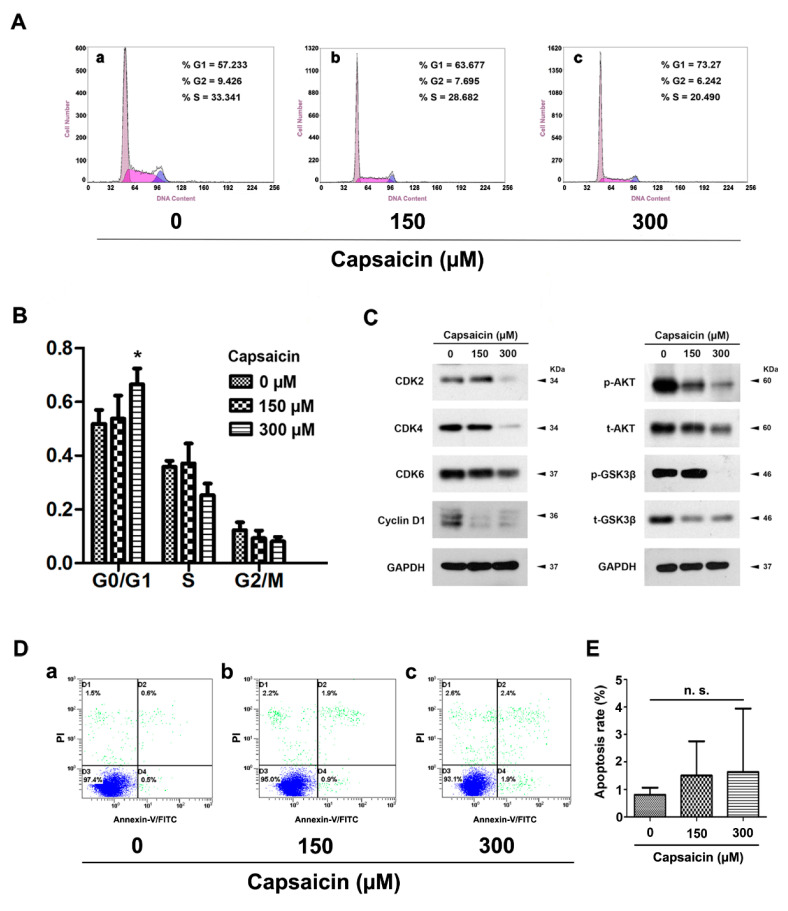
CAP triggered BCa cell cycle arrest but no significant effect on apoptosis in vitro. (**A**) Representative flow cytometry images for cell cycle alterations of CAP-treated 5637 cells at 0 (a), 150 (b) and 300 μM (c) for 48 h; (**B**) Statistical analysis of cell cycle alterations revealed a significant increased cell number at G0/G1 phase in the CAP-treated 5637 cells at 300 μM. All values shown were mean ± SD of triplicate measurements and repeated three times with similar results, * *p* < 0.05; (**C**) Western blot analysis revealed that the proteins involved in the G0/G1 phase regulation (CDK2, CDK4, CDK6, cyclin D1) and the PI3K/Akt/GSK3β signaling pathway (total and phosphorylated AKT/GSK3β) were all strongly downregulated after CAP treatment. GAPDH was used as a loading control. Cell types, concentrations of CAP treatment and protein masses were indicated; (**D**) Representative flow cytometry images for cell apoptosis of the CAP-treated 5637 cells at 0 (a), 150 (b) and 300 μM (c) for 48 h; (**E**) Statistical analysis of apoptotic rate in 5637 cell lines after CAP treatment, suggesting that no significant alteration of cell apoptosis was induced by CAP in the 5637 cells. All values shown were mean ± SD of triplicate measurements and repeated three times with similar results (n.s. = no significance).

Flow cytometry analysis was performed to evaluate alterations of cell cycle (Figure 3A) and apoptosis (Figure 3D) in the CAP-treated 5637 cells. Statistical analysis indicated that CAP treatment at 300 µM for 48 h could significantly induce cell cycle arrest at G0/G1 phase (Figure 3B). Proteins (CDK2/4/6 and cyclin D1) regulating the G0/G1 phase were consistently considerably decreased in the 300 µM group (Figure 3C). Furthermore, the expressions of proteins involved in the PI3K/Akt/GSK3β signaling pathway were altered as well, such as total and phosphorylated AKT/GSK3β (Figure 3C). Notably, we observed that CAP could not promote apoptosis in 5637 cells significantly by flow cytometry analysis (Figure 3D,E).

### 2.4. Injection of CAP Suppressed Tumor Growth In Vivo

**Figure 4:**
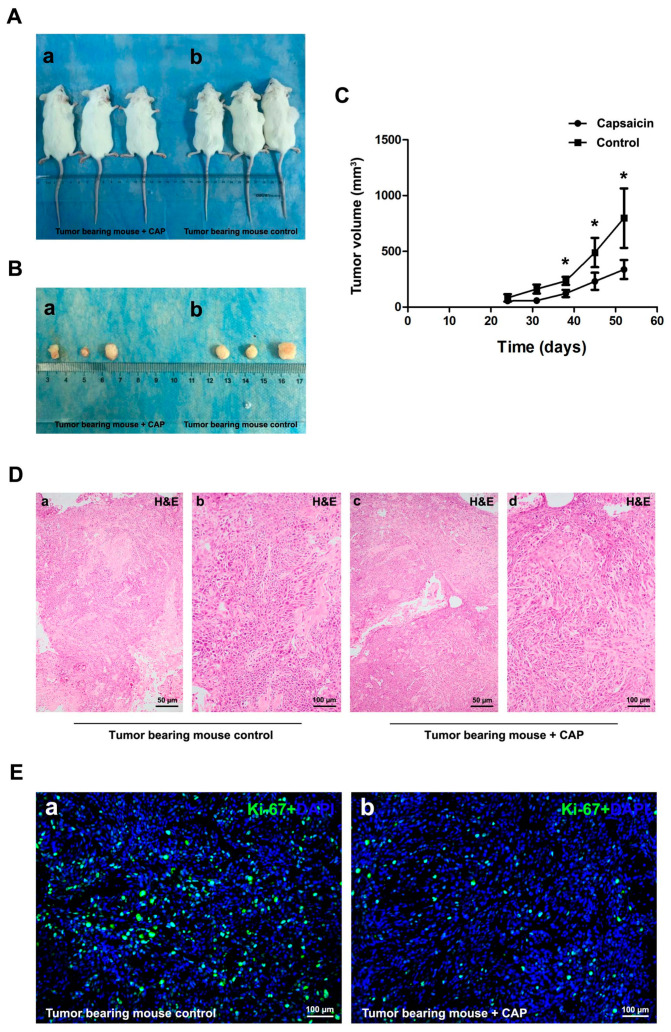
Delayed tumor growth after CAP treatment in vivo. (**A**) NOD/SCID mice were subcutaneously transplanted with 5637 cells for 21 days, and continuously injected into the peritumoral area by CAP for 28 days (a) compared with injection by saline containing 2.59% ethanol (b) as control; (**B**) Dissected tumor from the NOD/SCID mice injected by CAP (b) and saline containing 2.59% ethanol (a); (**C**) Statistical analysis of tumor size (mm^3^) measured by a caliper and calculated using *t*-test, * *p* < 0.05. Days after 5637 cells transplantation, tumor size and control (saline containing 2.59% ethanol)/CAP injection were indicated; (**D**) Representative H & E staining of the tumor bearing mice control (a,b) and CAP injection (c,d) tissues. The scale bar for (a,c) are 50 µm and for (b,d) are 100 µm; (**E**) Cell proliferation of 5637 cells in vivo treated by control (a) and CAP (b) was detected by Ki-67 immunofluorescence staining (green). Nuclei were stained by DAPI (blue). The scale bar for (a,b) are 100 µm.

To further evaluate the effects of CAP on the tumor growth in vivo, we established a NOD/SCID mouse model transplanted with 5637 BCa cells (Figure 4A,B). Our results showed that CAP injection could suppress tumor growth significantly (Figure 4C). The tissues from tumor bearing mice were embedded into paraffin and stained by Hematoxylin and Eosin. The staining results suggested the reduced number of tumor cells in the CAP-injected group (Figure 4D). Moreover, immune-fluorescence staining of Ki-67, an important marker for cell proliferation [28], exhibited considerably less Ki-67 positive BCa cells (green) in the CAP-injected group compared with the control group (Figure 4E).

### 2.5. CAP Interfered ROS Metabolism In Vivo

**Figure 5:**
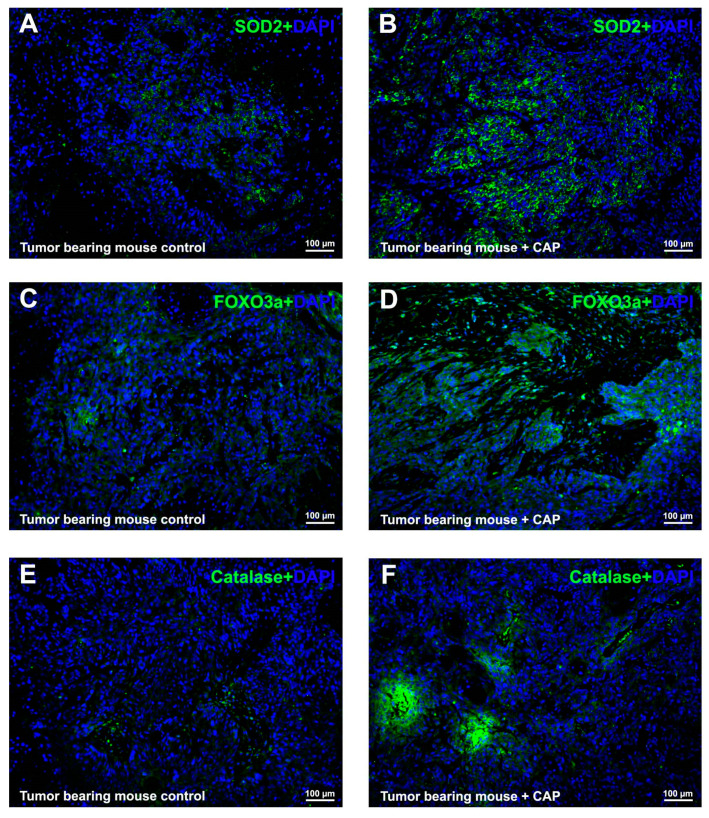
CAP induced strongly upregulation of proteins involved in ROS metabolism in vivo. Representative immunofluorescence staining revealed a strongly increasing of proteins involved in ROS metabolism, SOD2 (green, **A**,**B**); FOXO3a (green, **C**,**D**) and Catalase (green, **E**,**F**); in the tumor bearing mice control (**A**,**C**,**E**) and CAP injection (**B**,**D**,**F**) tissues. Nuclei were stained by DAPI (blue). The scale bar for (**A**–**F**) are 100 μm.

Immunofluorescence analysis revealed a strong increase in signal intensity for mitochondrial SOD2 (Figure 5B) and peroxisomal Catalase (Figure 5F) in the tissues from the CAP-injected tumor bearing mice, compared with the control group (Figure 5A,E).

Also, the transcriptional factor FOXO3a was considerably induced in vivo as well (Figure 5D), suggesting the disturbed cell cycle regulation and ROS metabolism under the influence of CAP could be via FOXO3a-mediated pathways.

## 3. Discussion

Recent studies revealed an anti-tumor effect of CAP on several human carcinoma cells, including bladder cancer cells [17], ovarian cancer cells [29], breast cancer cells [30], lung cancer [31], hepatocarcinoma cells [32], colon carcinoma cells [33], etc. However, the detailed mechanisms of CAP on cancer cells remain largely unknown. CAP could specifically activate TRPV1 [12,13] and interfere with the calcium signaling pathway [26]. Based on our previous transcriptome analysis studies comparing human bladder cancer tissues versus normal bladder tissues, a significant correlation between the calcium signaling pathway, FOXO signaling pathway, cell cycle regulation, PPARγ-related ROS metabolism and tumorigenesis of BCa was indicated [21,22,23]. Indeed, our results showed that CAP suppressed proliferation of BCa cells in a dose-dependent manner (0, 150 and 300 μM), which were also approximately consistent with previous studies [34,35].

Capsaicin was reported to suppress tumor growth primarily through induction of apoptosis [32,36,37]. However, we have found no significant effect of CAP on apoptosis of human bladder cancer 5637 cells in vitro (Figure 3). The occurrence of cell apoptosis induced by CAP treatment might be influenced by following reasons: firstly, purity of CAP used in the previous studies ranged from about 65% to >99% (in the case of synthetic pure CAP) [11]. Secondly, intrinsic diversities might exist in distinct cell lines. For instance, Zheng et al. [38] reported that activation of TRPV1 by CAP could strongly inhibit growth of 5637 cells (high expression of TRPV1), whereas growth of T24 cells (low expression of TRPV1) was not considerably suppressed. Thirdly, various adaptations might occur to cancer cell lines during CAP treatment [35,39]. Lewinska et al. [35] reported that CAP was unable to provoke apoptotic cell death when used up to 250 μM concentrations to treat human lung A549 cells and prostate cancer DU145 cells for 24 h. Furthermore, they found that genotoxic effects of CAP may contribute to limited susceptibility of DU145 and A549 cancer cells to apoptosis. In addition, Choi CH et al. [39] reported that CAP could induce apoptosis in nonmalignant human breast MCF10A cells, whereas a non-apoptotic cell cycle arrest in MCF7 and MDA-MB-231 breast cancer cells. Thus, their studies revealed that CAP might be associated with autophagy and play a key role in retardation of cell death by blocking CAP-induced endoplasmic reticulum (ER) stress-mediated apoptosis in MCF7 and MDA-MB-231 cells. In this context, CAP-induced autophagy could protect breast cancer cells against apoptosis. Taken together, CAP might have a potential dual effect on cancer cells, which might be dose-dependant and needed to be further investigated.

As reported by Lin et al., mitochondrial ROS generation could be the major source of ROS and therefore lead to CAP-induced apoptosis of bladder cancer TSGH cells [17]. In contrast, we observed no significant effect on apoptosis of human bladder cancer 5637 cells but a significantly induced cell cycle arrest at G0/G1 phase in the 5637 cells (Figure 3), followed by downregulation of proteins involved in G0/G1 to S phase progression (CDK2/4/6 and cyclin D1). Moreover, we also observed significant induction of ROS production in 5637 and T24 cells by DCFH-DA staining and flow cytometry analysis, respectively (Figure 2). In fact, the role of ROS in cancer biology is rather complex, which acts as a double-edged sword. A modest level of ROS is required for tumor promotion, while an excessive level serves to suppress tumors [40,41]. ROS may serve as an either survival or apoptotic signal, which depends on dosage, duration, type and site of ROS production [42].

Since our transcriptome analysis suggested a close link among cell cycle regulation, ROS metabolism and FOXO signaling pathway in bladder cancer, we analyzed alteration of transcriptional factor FOXO3a, a key subtype in the FOXO family involving in AKT/FOXO3a/β-catenin pathway [43,44,45] and playing a key role in regulating cell cycle, oxidative stress response and apoptosis [46,47,48]. Indeed, our in vitro (Figure 2) and in vivo (Figure 5) studies indicated a strong increase of FOXO3a in the CAP-treated bladder cancer cells, suggesting that the induction of cell cycle arrest and ROS production triggered by CAP could be via FOXO3a-mediated pathways. However, further studies are needed to clarify the significantly altered pathways, for example using map04068 (http://www.genome.jp/kegg-bin/show_pathway?map04068), KEGG pathway image, Kanehisa Laboratories, Japan [49,50].

Moreover, our results revealed strongly upregulation of peroxisomal catalase in the CAP-treated group. Catalase has been reported to protect chromosomes from oxidative damage [51] or ionizing radiation [52] and therefore could suppress cell death, which may be a reason that no significant increase of apoptosis occurred in the CAP-treated 5637 cells. Furthermore, as reported by Yang et al. from our group, CAP could induce cellular oxidative stress via mitochondrial ROS generation in bladder cancer T24 cell, whereas no obvious apoptosis took place in the CAP-treated T24 cells [26]. Therefore, we investigated alteration of mitochondrial SOD2, which could clear mitochondrial ROS and protect against cell death [53]. Indeed, our study showed a strongly upregulation of SOD2 as well, which may be another possibility to explain no significant increase of apoptotic cells observed.

In conclusion, our study revealed that CAP could suppress proliferation and induce cell cycle arrest as well as ROS production possibly via FOXO3a-mediated pathways in bladder cancer cells, whereas no obvious cell apoptosis under the protection of increased catalase and SOD2 enzymes. 

## 4. Experimental Section

### 4.1. Ethical Statement for Mice (NOD/SCID)

Investigation has been conducted in accordance with the ethical standards and according to the Declaration of Helsinki and according to national and international guidelines and has been approved by Ethic Committee at Zhongnan Hospital of Wuhan University (approval number: 02516036Z). Non-obese diabetic/severe combined immunodeficiency (NOD/SCID) male mice used in this study were purchased from Beijing HFK Bioscience Co., Ltd. (Beijing, China).

### 4.2. Human Bladder Cancer Cell Lines

Human bladder cancer cell lines T24 (transitional cell carcinoma, Cat. #SCSP-536) and 5637 (grade II carcinoma, Cat. #TCHu1) were kindly provided by the Stem Cell Bank, Chinese Academy of Sciences (Shanghai, China). Identification of the BCa cell lines was at the China Centre for Type Culture Collection (Wuhan, China). The BCa cells were cultured in RPMI-1640 medium (Gibco, Shanghai, China) containing 1% penicillin G sodium/streptomycin sulphate and 10% fetal bovine serum (FBS) (Gibco, Scoresby, Australia) in a humidified atmosphere consisting of 95% air and 5% CO_2_ at 37 °C.

### 4.3. Capsaicin Treatment for BCa Cells In Vitro

For in vitro study, BCa cells (5637 and T24) were cultured for 24 h and then treated by CAP (Cat. #ab141000, Abcam, Cambridge, UK) at 0, 50, 100, 150, 200 and 300 μM for 48 h. To prepare the culture media containing different concentrations of CAP, CAP was initially dissolved in DMSO to a concentration of 200 mM and subsequently dissolved in RPMI-1640 medium (Gibco, Shanghai, China) containing 10% fetal bovine serum (FBS; Gibco, Scoresby, Australia).

### 4.4. Xenograft Model

Before the experiments, the male NOD/SCID mice were adapted to the environment for a week. All mice were housed in a specific pathogen-free, temperature and humidity-controlled environment with food and water in their cages. 5637 cells (4 × 10^7^/mL in PBS) were injected in the right flank of the male NOD/SCID mice (*n* = 6) at day 35 subcutaneously (100 µL for each mouse). The mice were then feed for 21 days and the transplanted tumors were approximately at 50 mm^3^. CAP (20 mg per kg body weight) was injected to the mice (*n* = 3) into the peritumoral area every two days for four weeks. To prepare the injection solution, CAP was initially dissolved in ethanol to a concentration of 200 mM and subsequently dissolved in saline to a concentration of 2 mg/mL. The final concentration of ethanol was 2.59%. Saline containing 2.59% ethanol injected into the mice (*n* = 3) was used as a control. The tumor size for each mouse was measured every week using a caliper and calculated using the formulation as: tumor volume (mm^3^) = π/6 × length × width^2^ [54]. No skin ulcers were observed.

### 4.5. Cell Culture Experiments

#### 4.5.1. MTT Test for CAP Treatment

MTT assay was used to analyze the cell viability. One hundred µL cell suspensions (2 × 10^4^ cells per mL) with 10% FBS medium was seeded to a 96-well plate and incubated (37 °C and 5% CO_2_) for overnight. After treatment with different concentrations of CAP, 10 µL MTT (5 mg/mL) reagent was added to each well at the indicated time and incubated for 4 h at 37 °C. Remove the media and add 150 µL of DMSO to each well. Absorbance was measured at 490 nm by a microplate reader (Cat. #SpectraMax M2, Molecular Devices, Sunnyvale, CA, USA). MTT assay results were reported as Relative cell proliferation values. The absorbance value of each measurement was normalized to the value of CAP at 0 µM (DMSO control) for each concentration of CAP, calculated as: Relative cell proliferation = MTT Absorbance value of CAP-treated cells/MTT absorbance value of CAP-untreated cells.

#### 4.5.2. Transwell Migration Assay

A 24-well plate transwell chamber system (Corning, Glendale, CA, USA) with 8.0 µm pore size was used. Cells were suspended in 0.5% FBS medium at a density of 5 × 10^5^ cell/mL and 100 µL cell suspension was seeded in the upper chamber, while the lower chamber was filled with 10% FBS medium. After 24 h incubation at 37 °C, cells on the upper insert were removed by cotton swabs, and cells that migrated to the lower side were fixed with formalin for 30 min and stained with crystal violet. Then the chambers were placed under an inverted phase contrast microscope and 16 random areas were selected to observe and count the migrated cells.

#### 4.5.3. Flow Cytometry Analysis for Alterations of Cell Cycle and Apoptosis

For cell cycle analysis, 1 × 10^6^ cells were harvested and fixed in 70% ice cold ethanol at −20 °C for overnight. After centrifugation, pellets were resuspended with PBS containing 50 µg/mL propidium iodide (Sigma-Aldrich, St. Louis, MO, USA) and 0.1 mg/mL RNaseA (20 μg/mL in PBS) in the dark. After incubation at 37 °C for 30 min, the DNA content distribution was analyzed by flow cytometry analysis (Cat. #FC500, Beckman Coulter, Brea, CA, USA). For apoptosis analysis, after treatment with CAP for 48 h, cells were harvested, washed with PBS, and stained with FITC Annexin V Apoptosis Detection Kit I (BD Biosciences, Franklin Lakes, NJ, USA) and analyzed by the flow cytometry analysis.

#### 4.5.4. ROS Detection by Staining with DCFH-DA

The fluorescent probe 2′,7′-dichlorofluorescin diacetate (DCFH-DA) was used to evaluate intracellular ROS levels. BCa cells treated by CAP and DMSO (control) were used for this experiment. After treatment with CAP at 0, 150 and 300 µM for 48 h, 5637 and T24 cells were harvested and incubated in the dark with 10 µM DCFH-DA prepared in serum-free RPMI-1640 medium at 37 °C for 30 min. Thereafter, the cells were washed three times with PBS and submitted to flow cytometry analysis. Relative fluorescence of ROS was determined by the ratio between the fluorescence of ROS value for CAP treated cells and the fluorescence of ROS value for control cells (DMSO).

For ROS staining, slides with the BCa cells were stained by DCFH-DA(10 µM) and incubated for 30 min at 37 °C in the dark, then washed by PBS three times. Nuclei were counterstained with 1 µM DAPI for 20 min at room temperature. DCFH-DA positive cells were counted per 100 random cells three times for statistical analysis. Images were taken with a fluorescence microscope (Cat. #IX73, Olympus, Tokyo, Japan).

### 4.6. Protein Analyses

#### 4.6.1. Isolation of Total Protein from BCa Cells and Western Blot Analysis

5637 cells and T24 cells were lysed and sonicated in RIPA buffer containing protease inhibitor and phosphatase inhibitor (Sigma-Aldrich) on ice for 30 min, then centrifuged at 12,000× *g* for 15 min to collect supernatant. By Bradford protein assay (Bio-Rad, Hercules, CA, USA) the concentrations of protein were determined using bovine serum albumin (BSA) as standard. The isolated total protein was loaded using 10%–12.5% SDS-PAGE and transferred to PVDF membrane (Millipore, Billerica, MA, USA). Membranes were blocked by 5% non-fat milk for about 2 h and incubated with primary antibodies (Table 1) at 4 °C for overnight. After washing four times each for 10 min, the membranes were incubated with secondary antibody (listed in Table 2) at room temperature for about 2 h. Bands were visualized using an enhanced chemiluminescence (ECL) kit (Bio-Rad) and detected by Biomax MR films (Kodak, Rochester, NY, USA).

#### 4.6.2. Immunofluorescence Staining for Xenograft Mouse Tissues

All the transplanted tumor samples were fixed by 4% PFA at 4 °C overnight and embedded into paraffin (Paraplast, Sigma-Aldrich) using tissue processor (Cat. #STP 120 and Cat. #Histostar, Thermo Fisher Scientific, Loughborough, UK). Paraffin sections (5 µm) were cut with a rotation microtome (Cat. #HM325, Thermo Fisher Scientific, Bremen, Germany). The sections were serially incubated with indicated primary antibody (listed in Table 1) and Cy3-labeled or FITC-labeled secondary antibody (listed in Table 2) in humidified atmosphere. Nuclei were labeled with DAPI (2 μg/mL). Sections were analyzed by fluorescence microscope.

#### 4.6.3. Hematoxylin and Eosin (H & E) Staining

Paraffin sections (5 µm thick) of transplanted tumor tissues from NOD/SCID mice were stained with Hematoxylin and Eosin. Sections were deparaffinized and rehydrated by xylene 3 × 10 min, 100% ethanol 2 × 5 min, 96% ethanol, 80% ethanol, 70% ethanol, and H_2_O, 5 min for each step. The sections were stained for 7 min in 10% Hematoxilin (Sigma-Aldrich). After washing 10 min under the tap water for revealing the nuclei, the cytoplasm was stained for 5 min in 1% Eosin (Sigma-Aldrich) containing 0.2% glacial acetic acid. The slides were shortly washed with tap water and dehydrated short in 1 × 70%, 1 × 80%, 2 × 96%, 3 × 100% ethanol, 2 min for each step, followed by 3 × 10 min in xylene. The sections were imaged by an inverted phase contrast microscope (Cat. #DMI 1, Leica, Wetzlar, Germany).

### 4.7. Statistical Analyses

Data were expressed as mean ± SD from at least three independent experiments. All analyses were performed three times and represent data from three individual experiments. Two-tailed Student’s *t*-tests were used to evaluate the statistical significance of differences of the data. All of the statistical analyses were performed with SPSS 16.0 (SPSS Inc., Armonk, NY, USA). The statistical significance was set at probability values of *p* < 0.05.

## Figures and Tables

**Table 1:** List of primary antibodies.

Antigens	Species Antibodies Raised in	Dilution (IF)	Dilution (WB)	Supplier
Akt (pan), mouse	Rabbit, monoclonal	-	1:2000	Cell Signaling Technology, Danvers, MA, USA, Cat. #4691
CDK2, human	Rabbit, monoclonal	-	1:2000	Cell Signaling Technology, Cat. #2546
CDK4, human	Rabbit, monoclonal	-	1:2000	Cell Signaling Technology, Cat. #12790
CDK6, human	Rabbit, monoclonal	-	1:1000	Abcam, Cat. #ab124821
Cyclin D1, human	Rabbit, monoclonal	-	1:2000	Cell Signaling Technology, Cat. #2978
*E*-cadherin, human	Rabbit, monoclonal	-	1:500	Cell Signaling Technology, Cat. #3195
Glyceraldehyde 3-phosphate dehydrogenase (GAPDH), human	Mouse, monoclonal	-	1:2000	Santa Cruz Biotechnology Inc., Dallas, TX, USA, Cat. #sc-365062
*N*-Cadherin, human	Rabbit, monoclonal	-	1:1000	Cell Signaling Technology, Cat. #13116
β-Catenin, human	Rabbit, monoclonal	-	1:1000	Cell Signaling Technology, Cat. #8480
*p*-AKT(Thr308), human	Rabbit, polyclonal	-	1:1000	Cell Signaling Technology, Cat. #9275
Vimentin, human	Rabbit, monoclonal	-	1:2000	Cell Signaling Technology, Cat. #5741
Ki-67, human	Rabbit, polyclonal	1:200	-	Novus Biologicals, Littleton, CO, USA, Cat. #NBP2-19012
Catalase, human	Rabbit, monoclonal	1:200	1:2000	Abcam, Cat. #ab76024
SOD2, human	Rabbit, monoclonal	1:200	1:1000	Abcam, Cat. #ab68155
FOXO3a, human	Rabbit, monoclonal	1:200	1:1000	Abcam, Cat. #ab53287
*t*-GSK-3β, human	Rabbit, monoclonal	-	1:15,000	Cell Signaling Technology, Cat. #12456
*p*-GSK-3β, human	Rabbit, monoclonal	-	1:15,000	Cell Signaling Technology, Cat. #5558

WB: Western blot. IF: immunofluorescence staining.

**Table 2:** List of secondary antibodies and counterstaining of nuclei.

Secondary Detection System Used	Host	Method	Dilution	Supplier
Anti-Mouse-IgG (H + L)-HRP	Goat	WB	1:10,000	Sungene Biotech, Tianjin, China, Cat. #LK2003
Anti-Rabbit-IgG (H + L)-HRP	Goat	WB	1:10,000	Sungene Biotech, Cat. #LK2001
Anti-rabbit IgG (H + L), F(ab′)2 Fragment (Alexa Fluor^®^ 488 Conjugate)	Goat	IF	1:50	Cell Signaling Technology, Cat. #4412
Hoechst 33342 nucleic acid staining (DAPI)	-	IF	1:750	Molecular Probes/Invitrogen, Carlsbad, CA, USA, Cat. #A11007

WB: Western blot. IF: immunofluorescence staining.
