# Correction: Qian et al. Capsaicin Suppresses Cell Proliferation, Induces Cell Cycle Arrest and ROS Production in Bladder Cancer Cells through FOXO3a-Mediated Pathways. *Molecules* 2016, *21*, 1406

**DOI:** 10.3390/molecules27196731

**Published:** 2022-10-09

**Authors:** Kaiyu Qian, Gang Wang, Rui Cao, Tao Liu, Guofeng Qian, Xinyuan Guan, Zhongqiang Guo, Yu Xiao, Xinghuan Wang

**Affiliations:** 1 Department of Urology, Zhongnan Hospital of Wuhan University, Wuhan 430071, China; 2 Department of Urology, The Fifth Hospital of Wuhan, Wuhan 430050, China; 3 Department of Urology, Jingzhou Central Hospital, Jingzhou 434020, China; 4 Department of Endocrinology, The First Affiliated Hospital of Zhejiang University, Hangzhou 310003, China; 5 Department of Clinical Oncology, Li Ka Shing Faculty of Medicine, University of Hong Kong, Hong Kong, China; 6 Center for Medical Science Research, Zhongnan Hospital of Wuhan University, Wuhan 430071, China

During the course of a review of our publications, an inadvertent error in the article [1] has come to our attention. The authors regret that the data was incorrectly displayed in Figure 1Ce, and we wish to correct this error. The correct version of Figure 1C is as follows (high resolution image is in the attachment):

The error does not change the conclusion or interpretation of our results. Nevertheless, we consider it necessary to correct the misplaced image.

The authors would like to apologize for any inconvenience caused.

## Figures and Tables

**Figure 1:**
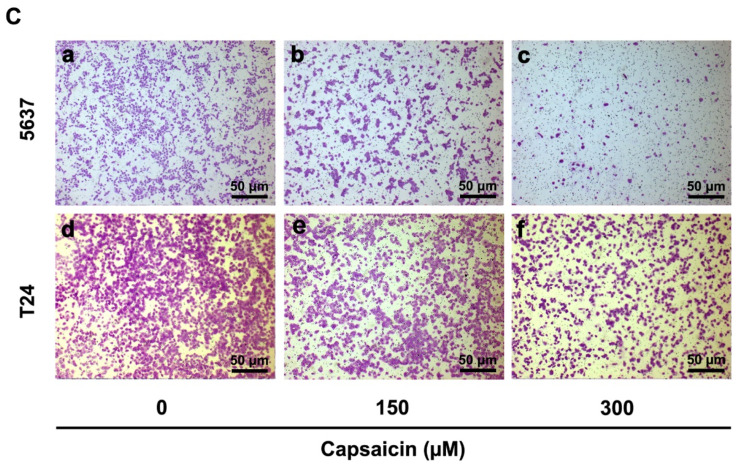
(**C**) Transwell migration assay for CAP treated 5637 (**a**–**c**) and T24 cells (**d**–**f**) at 0, 150 and 300 µM for 48 h. The scale bar for (**a**–**f**) is 50 µm.
